# Supplementary material for: Insights into evolution and coexistence of the colibactin- and yersiniabactin secondary metabolite determinants in enterobacterial populations
Source: Microb Genom. 2021 Jun 15;7(6):000577. doi: 10.1099/mgen.0.000577 (PMC8461471; doi:10.1099/mgen.0.000577)
Supplement: Supplementary material 1 [file mgen-7-0577-s001.pdf]

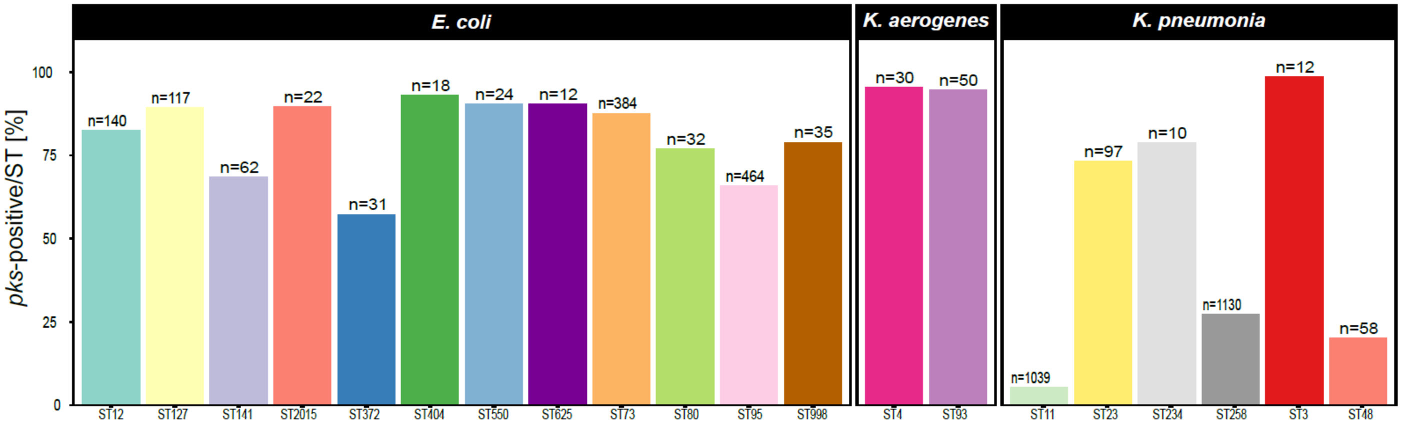

Figure S1. Sequence type distribution of *clb*-positive *E. coli* and *Klebsiella* strains.

We compared the amount of strains identified to harbor the *clb* gene cluster with the total number of strains belonging to their respective sequence types in our strain set. The absolute number of strains belonging to sequence types is shown on top of the bars.

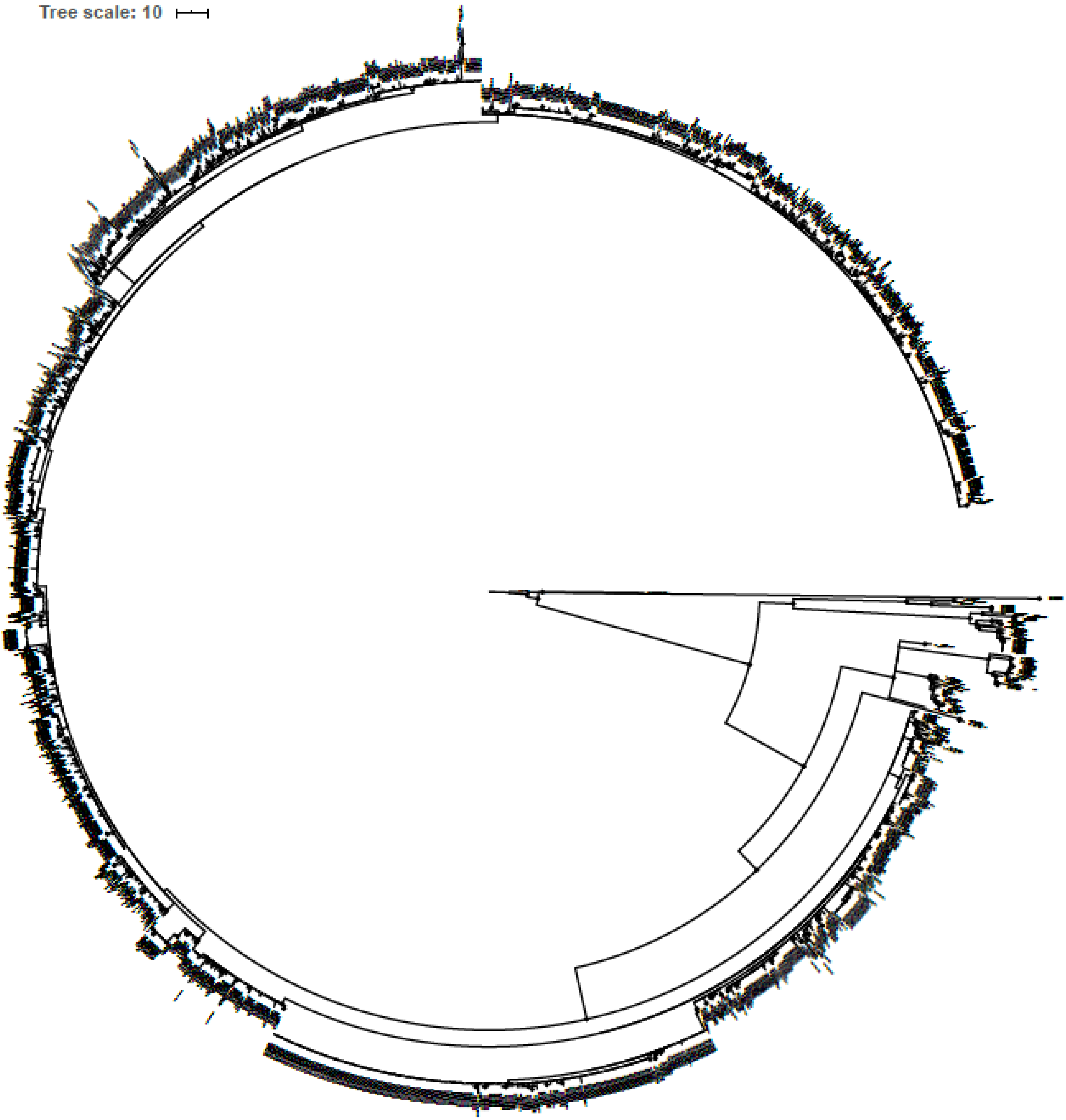

Figure S2. Maximum-likelihood based phylogeny of the colibactin gene cluster.

This phylogenetic tree represents all detected colibactin gene clusters in 2169 enterobacterial genomes (included also in Fig. 2A and Fig. 3), including strain labels and branch support values.

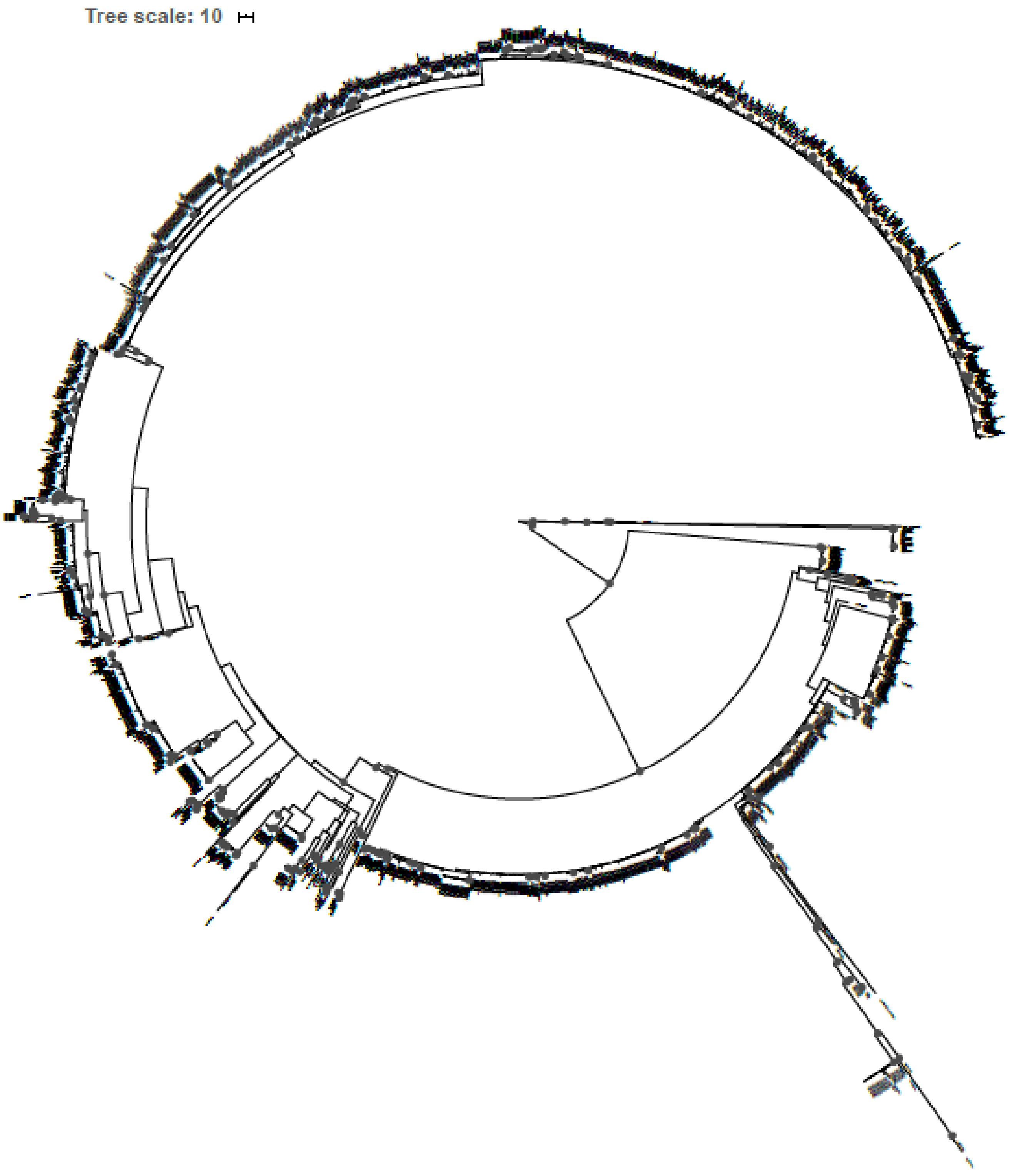

Figure S3. Maximum-likelihood based phylogeny of the yersiniabactin gene cluster.

This phylogenetic tree represents all yersiniabactin gene clusters as detected in colibactin positive enterobacterial genomes (included also in Fig. 2B), including strain labels and branch support values.

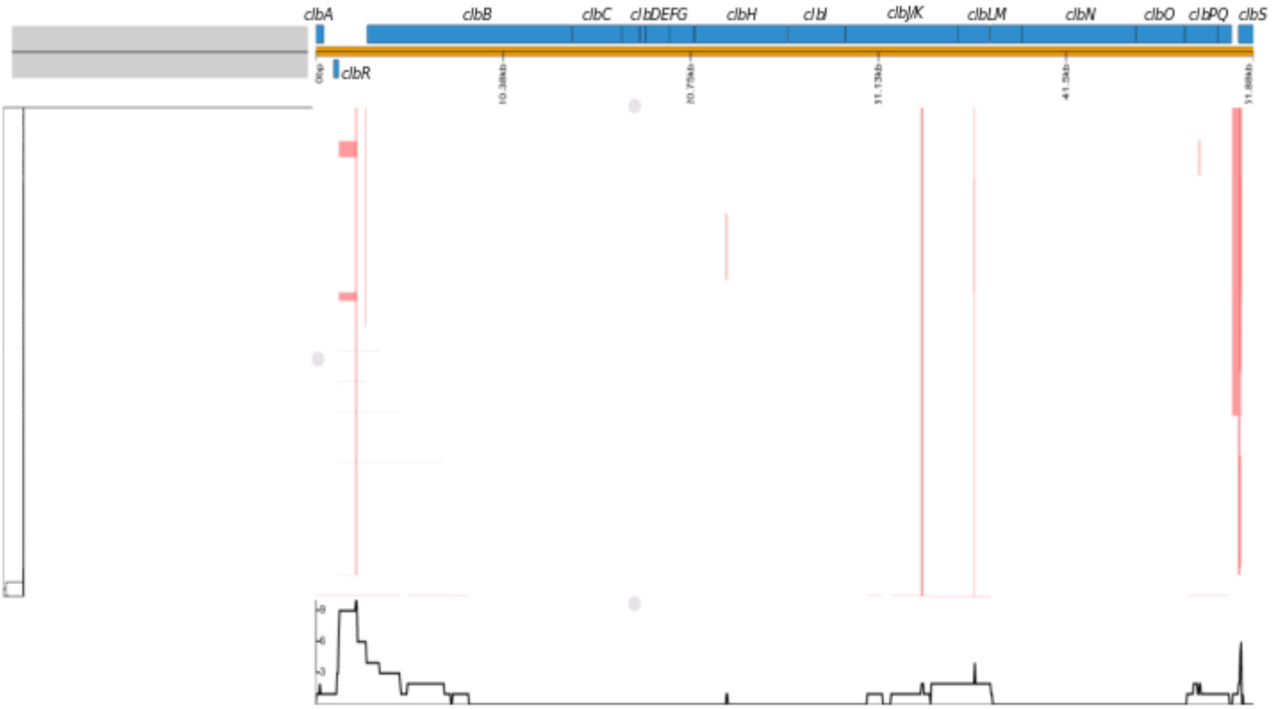

Figure S4. Predicted recombination events within the colibactin gene cluster.

Recombination events predicted by Gubbins within the colibactin gene cluster, alongside the phylogeny as shown on the left. The colibactin genes found in the reference genome used (*E. coli* strain M1/5) are shown with blue blocks. The predicted sites of recombination are shown in red blocks.

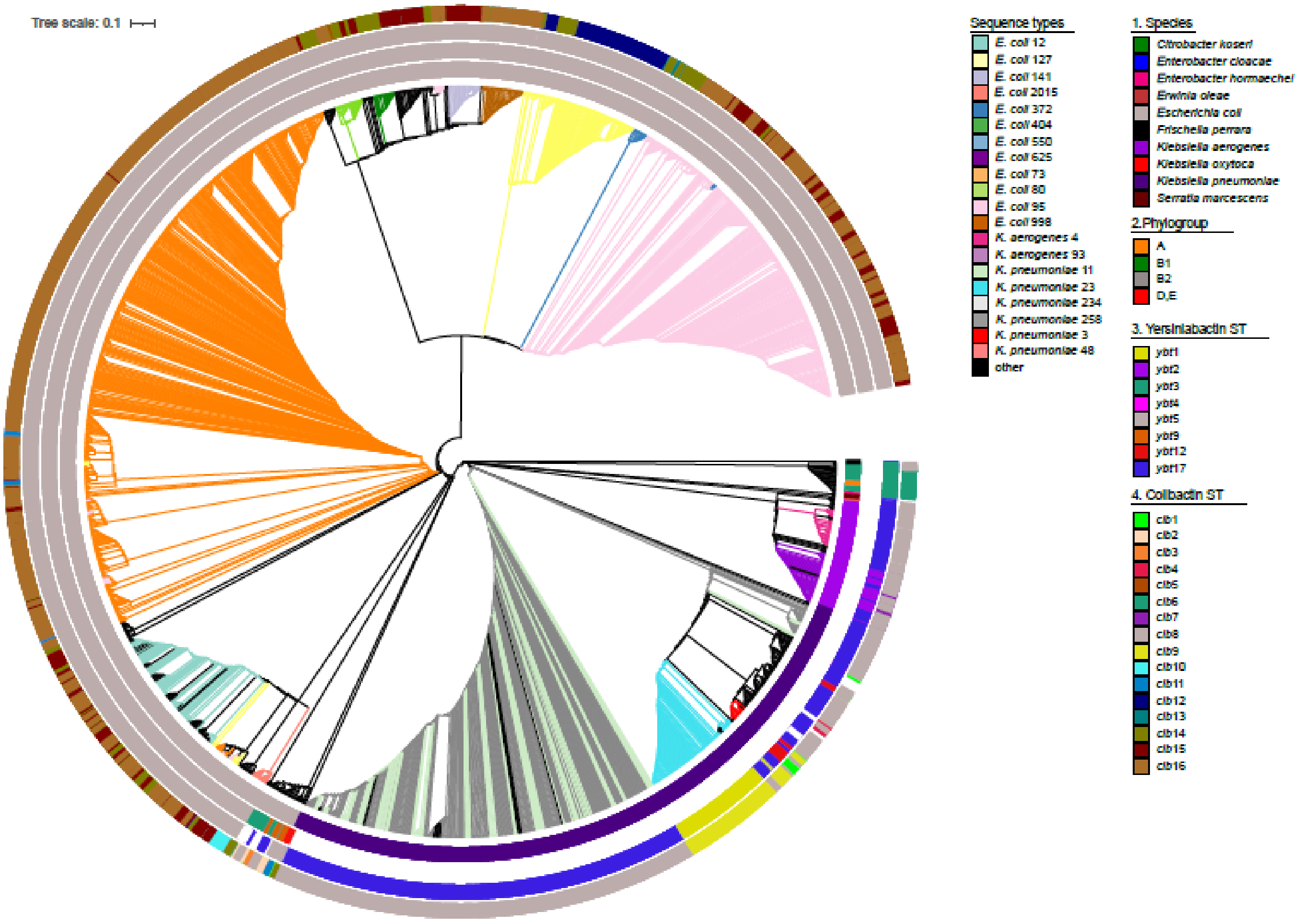

Figure S5. Core-genome-based phylogeny of the Enterobacterales strains included into the analysis. This phylogenetic tree is based on core genome sequence data described by Adeolu et al., 2016. From innermost to outermost, the 1st circle indicates the species harboring the clb determinant; the 2nd circle shows the E. coli phylogroup, the 3rd circle shows the yersiniabactin sequence types (YbST) of the *ybt* determinant. The 4th circle shows the different colibactin sequence types (ClbST) of the *clb* gene cluster. The branch colors in the center of the tree depict the prominent bacterial sequence types. Branch lengths (shown on Fig. S6) have been omitted.

[**Adeolu M, Alnajar S, Naushad S, R SG**. Genome-based phylogeny and taxonomy of the '*Enterobacteriales*': proposal for *Enterobacterales* ord. nov. divided into the families *Enterobacteriaceae*, *Erwiniaceae* fam. nov., *Pectobacteriaceae* fam. nov., *Yersiniaceae* fam. nov., *Hafniaceae* fam. nov., *Morganellaceae* fam. nov., and *Budviciaceae* fam. nov. *Int J Syst Evol Microbiol* 2016;66(12):5575-5599.]

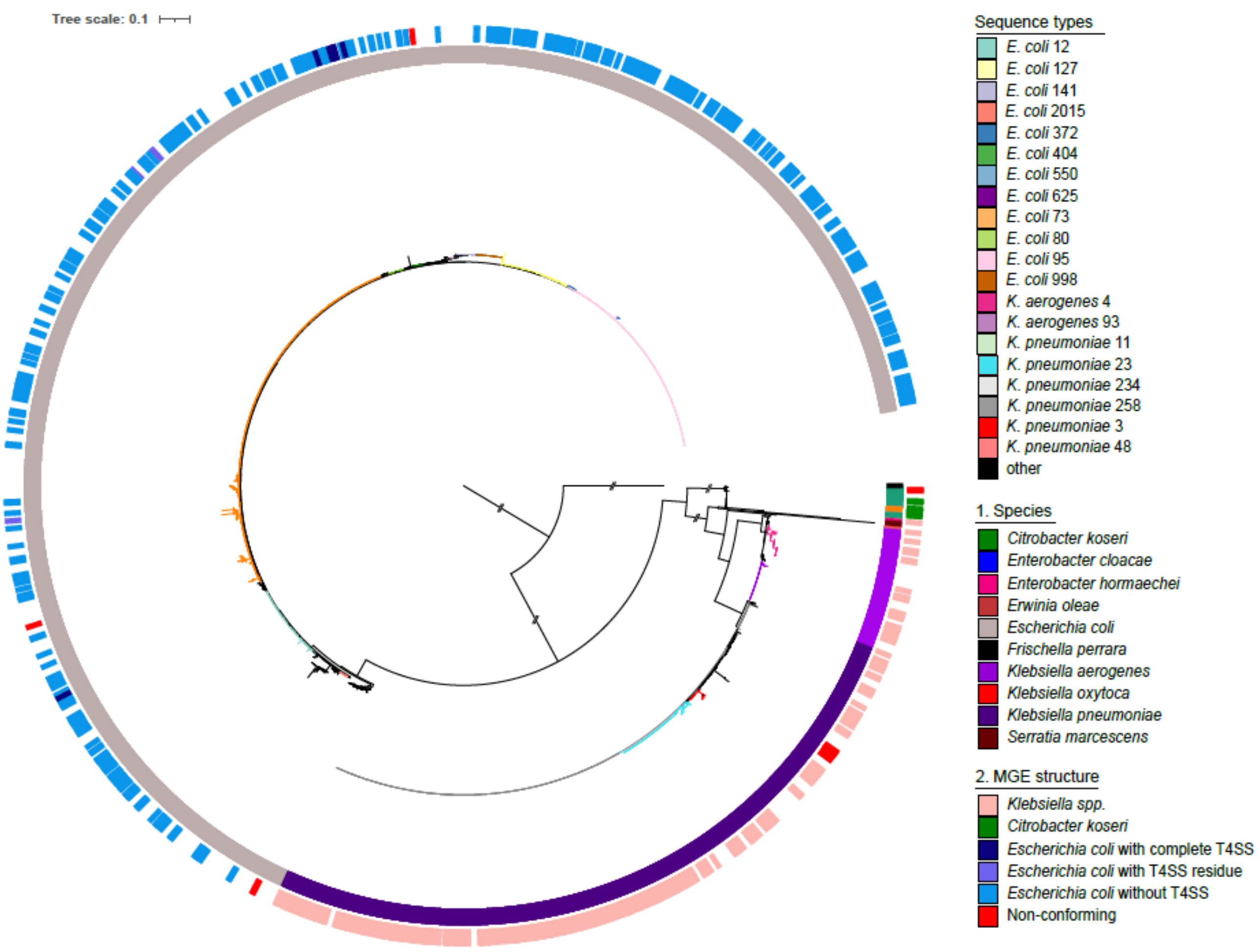

Figure S6. Correlation of *clb*-carrying MGE classes and phylogeny of the *Enterobacterales* strains included into the analysis.

This phylogenetic tree is based on core genome sequence data described by Adeolu et al., 2016. From innermost to outermost, the 1st circle indicates the species harboring the *clb* determinant; the 2nd circle shows the different species-specific MGE classes that have been described in Fig. 4 and Fig. 7. The MGEs that do not conform to the major structures (Fig. S7) are shown in red. The branch colors in the center of the tree depict the prominent bacterial sequence types.

[**Adeolu M, Alnajar S, Naushad S, R SG**. Genome-based phylogeny and taxonomy of the '*Enterobacteriales*': proposal for *Enterobacterales* ord. nov. divided into the families *Enterobacteriaceae*, *Erwiniaceae* fam. nov., *Pectobacteriaceae* fam. nov., *Yersiniaceae* fam. nov., *Hafniaceae* fam. nov., *Morganellaceae* fam. nov., and *Budviciaceae* fam. nov. *Int J Syst Evol Microbiol* 2016;66(12):5575-5599.]

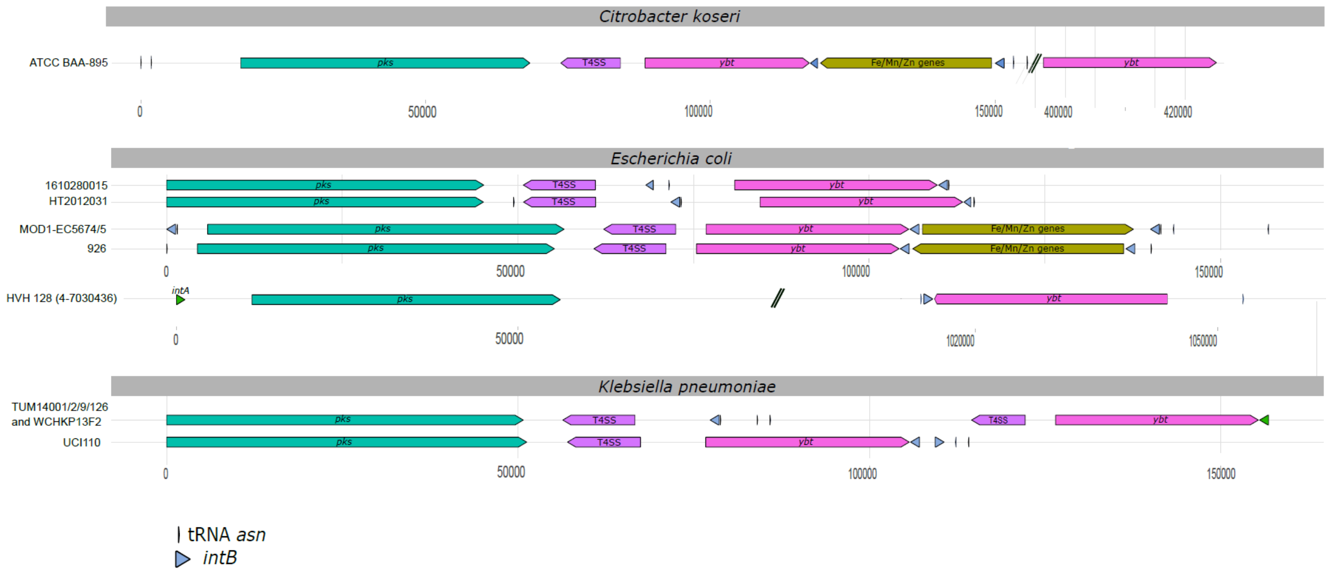

Figure S7. Structural variation of the *clb* and *ybt* gene cluster-harboring ICEs in *E. coli*, *K. pneumoniae*, and *C. koseri*.

The different structures found within ICEs that do not conform to the major structures shown in Fig. 4 are depicted. The *clb* gene cluster (teal green), T4SS module (purple), *ybt* determinant (pink), integrase genes (green/blue), and the Fe/Mn/Zn module (yellow) found within the ICE are shown.

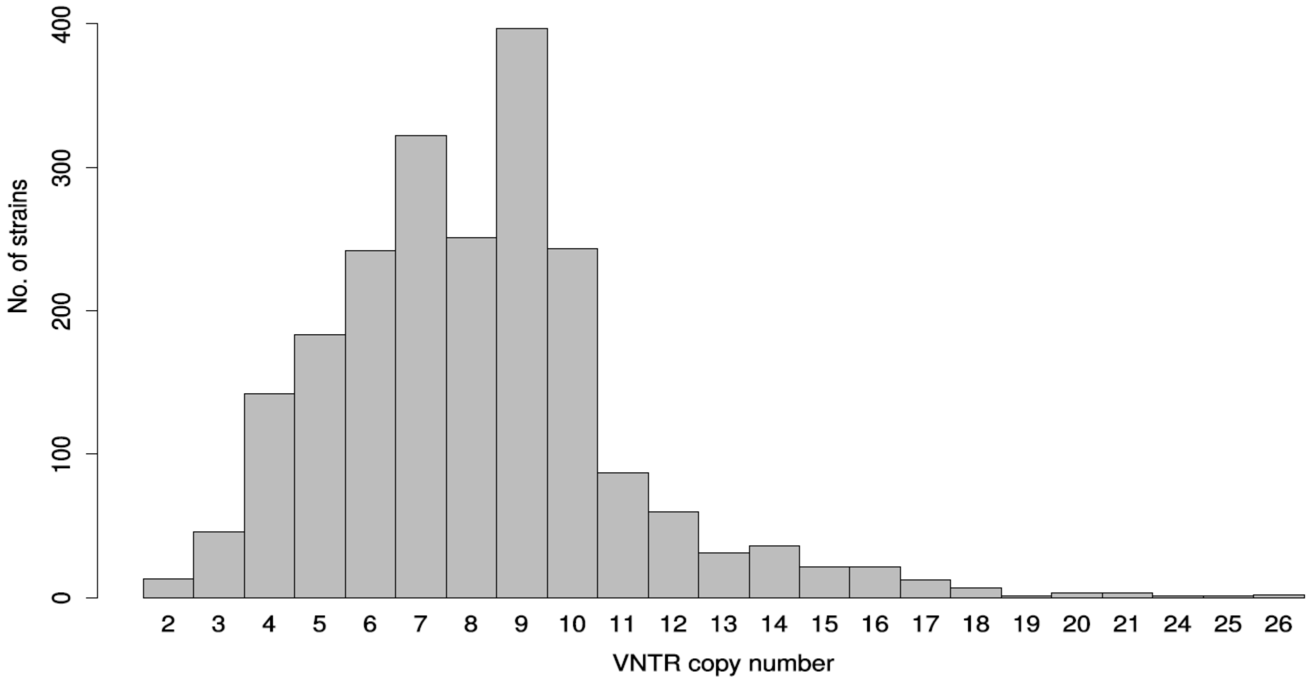

Figure S8. Size distribution of the variable number of tandem repeats (VNTR) region in *clb*-positive bacterial strains.

The number of repeats detected in the VNTR region located between *clbR* and *clbB* is shown in relation to the frequency of their occurrence in bacterial genomes.

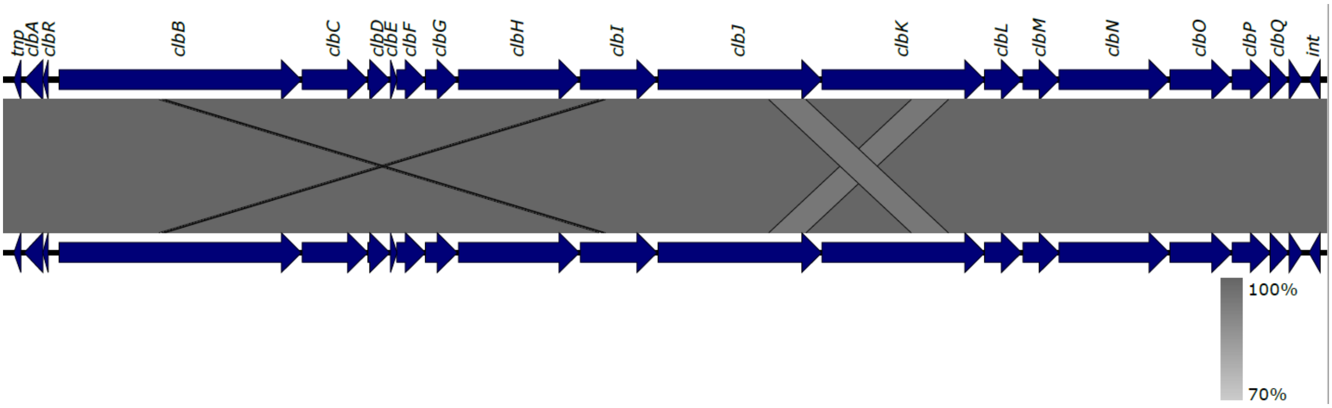

Figure S9. Linear comparison of the nucleotide sequence of the colibactin gene cluster.

Comparison of the colibactin genes found in *E. coli* strain M1/5 against themselves using Easyfig [Sullivan et al., 2011]. The alignment of colibactin genes, with a minimum nucleotide identity of 70%, shows the homology between regions of *clbJ* and *clbK*, which was discussed to be a potential cause for misassembly in short-read sequencing data.

[Sullivan MJ, Petty NK, Beatson SA. Easyfig: a genome comparison visualizer. Bioinformatics, Research Support, Non-U.S. Gov't 2011;27(7):1009-1010.]

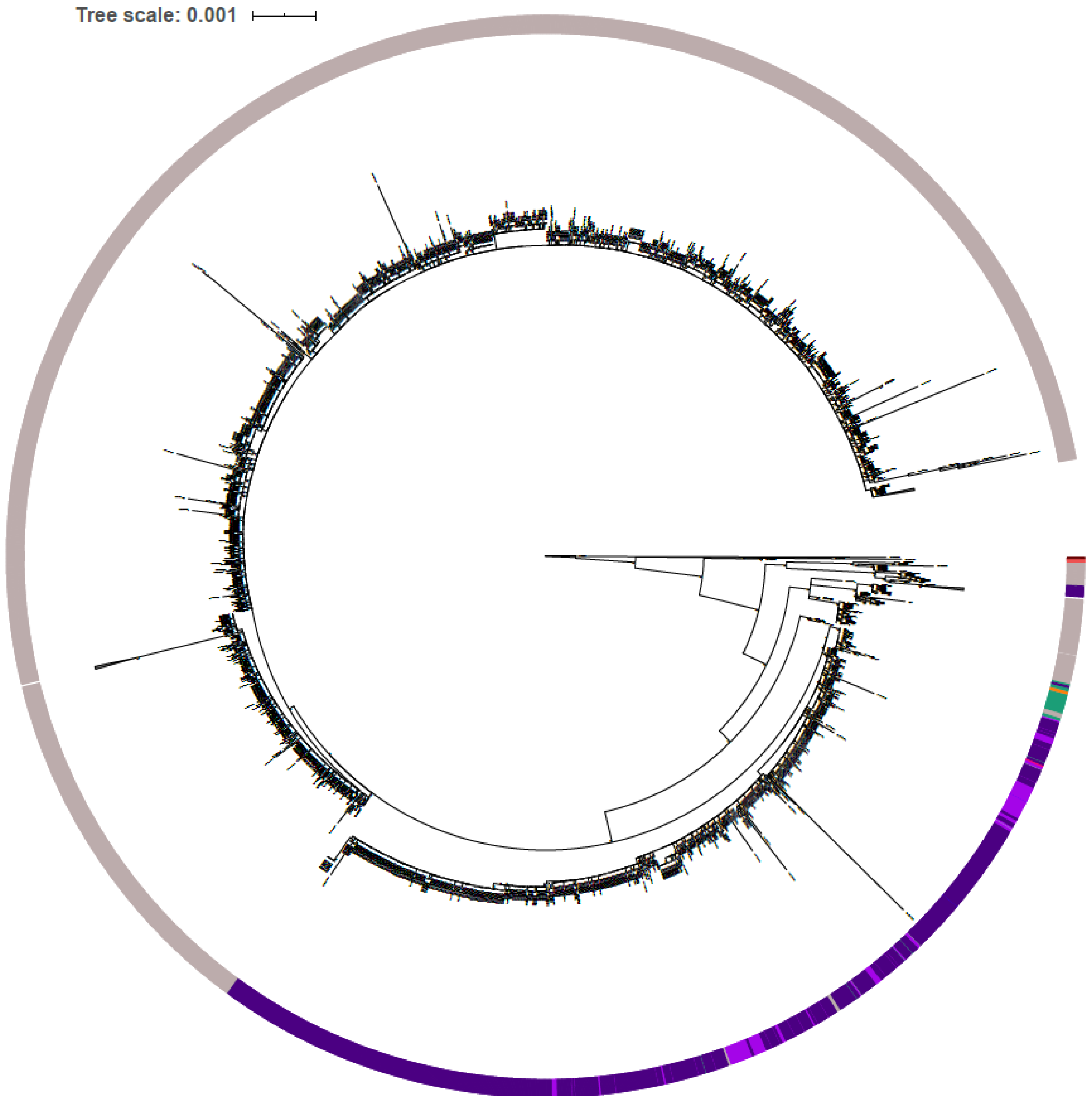

Figure S10. Phylogeny inferred from amino acid sequences of 17 *clb* genes. The amino acid sequences from colibactin genes were concatenated and used to generate a phylogeny in a similar manner to that of the nucleotide-based phylogeny. The outer circle describes the species of each node/strain harboring the colibactin genes.
